# A systematic review of geographic differences in knee phenotypes based on the coronal plane alignment of the knee (CPAK) classification

**DOI:** 10.1186/s42836-025-00311-4

**Published:** 2025-05-08

**Authors:** Guanghui Zhao, Chengyuan Ma, Zifan Luo, Jianbing Ma, Jianpeng Wang

**Affiliations:** Department of Joint Surgery, Honghui Hospital, Xi’an Jiaotong University, Xi’an, 710054 China

**Keywords:** Geographic differences, Coronal plane alignment knee classification, CPAK, Knee phenotypes, Total knee arthroplasty

## Abstract

**Background:**

The extent of geographic variation in knee phenotypes remains insufficiently documented. This systematic review intends to elucidate the regional disparities in the distribution of Coronal Plane Alignment of the Knee (CPAK) types across different geographic areas.

**Methods:**

A systematic review of the literature was conducted in adherence to the Preferred Reporting Items for Systematic Reviews and Meta-Analyses (PRISMA) guidelines. Studies reporting the distribution of knee phenotypes, as classified by the CPAK system, in both healthy and arthritic populations, were included in the analysis. Based on the methods in the literature, the Hoy Risk of Bias Tool was used to assess the methodological quality of the included studies. To compare geographical differences in CPAK types among patients with arthritis, as well as healthy people.

**Results:**

A total of 29 studies (28 retrospective and 1 prospective) were included in this review, encompassing 27,660 knees in 22,342 subjects. The methodological quality of the included studies was assessed using the Hoy Risk of Bias Tool, and the quality was good. Among the healthy knees (*n* = 4,082), CPAK type II was predominant in Europe (41.7%) and Asia (36.7%). In contrast, among arthritic knees (*n* = 21,632), CPAK type I was most common in Asia (51.3%), North America (32.8%), and Europe (32.8%), while CPAK type II was prevalent in Australia (29.3%) and Africa (25.5%). Notably, the proportions of CPAK type I (*P* < 0.001) and II (*P* = 0.002) knees varied significantly across different geographic regions among arthritic knees, while no significant differences were observed among healthy knees (*P* = 0.48, *P* = 0.305).

**Conclusion:**

Significant variations in CPAK distributions among arthritic knees were observed across countries, while no significant differences were observed among healthy knees. Surgeons in different regions need to make individual surgical plans according to the CPAK types of patients.

Video Abstract

**Supplementary Information:**

The online version contains supplementary material available at 10.1186/s42836-025-00311-4.

## Background

For decades, achieving a neutral mechanical alignment (hip-knee-ankle angle, HKA = 0°) has been a universal objective for orthopedic surgeons performing total knee arthroplasties (TKAs) [1]. Nonetheless, this strategy overlooked the anatomy of the native joint and the biomechanical interplay between the origins and insertions of the soft tissues crossing the joint, which has contributed to documented patient dissatisfaction following TKA [2]. Given the significant mismatches arising from the normal anatomical variability in native joint anatomy, there is a burgeoning interest in personalized alignment techniques during TKA, which aim to reinstate a patient’s constitutional (pre-arthritic) alignment [3, 4].

**Fig. 1:**
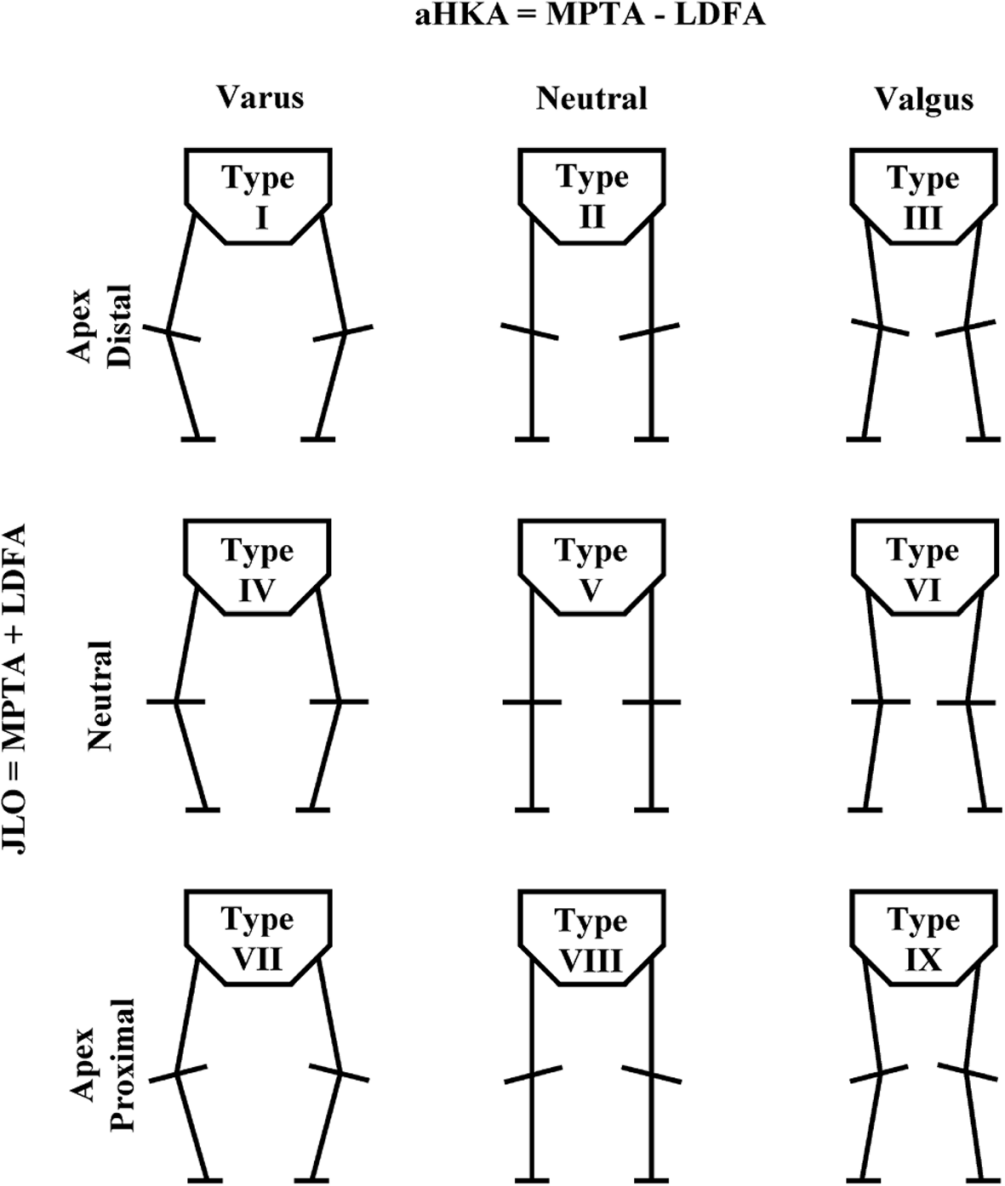
Coronal Plane Alignment of the Knee classification (CPAK) with nine theoretical types of the knee

In 2021, MacDessi et al. [5] introduced the Coronal Plane Alignment of the Knee (CPAK) classification system, which categorizes knee phenotypes into nine distinct types based on two primary criteria: constitutional limb alignment and joint line obliquity (JLO) (Fig. 1). Constitutional limb alignment is classified as varus, neutral, or valgus, represented by the arithmetic hip-knee-ankle angle (aHKA), calculated as medial proximal tibial angle (MPTA) minus lateral distal femoral angle (LDFA). JLO is characterized as apex distal, neutral, or apex proximal, determined by the sum of MPTA and LDFA. Since its introduction, this classification system has been widely adopted by researchers [6–8].

Comprehending geographic differences in knee phenotypes is essential for surgeons to tailor and enhance arthroplasty care. In 2023, Pagan et al. [9] conducted a systematic review to assess disparities in lower limb alignment across diverse countries/regions and revealed significant differences in CPAK distributions among these populations. However, the study’s scope was limited to seven studies, potentially unable to including the full spectrum of regional variations. In recent years, with the increasing adoption of the CPAK system in research, a richer dataset has emerged, enabling more comprehensive comparisons of lower limb alignment differences among populations from various countries/regions [7, 8].

This study conducted a systematic review to investigate and describe geographic disparities in CPAK types among healthy and arthritic knees.

## Material and methods

### Literature search

This systematic review adhered to Preferred Reporting Items for Systematic Reviews and Meta-Analyses (PRISMA) guidelines and was registered on PROSPERO (registration number: CRD42024613481) [10]. A thorough title and abstract search was conducted in PubMed, Embase, and the Cochrane Library, covering the period from the introduction of the CPAK classification in February 2021 to October 2024. The search utilized the terms “Coronal Plane Alignment of the Knee” or “CPAK” without language restriction. Inclusion criteria for studies were: (1) the observed population included healthy and/or arthritic knees without exclusion of specific patient groups, (2) the CPAK classification was applied following standardized protocols as outlined by MacDessi et al. [5], and (3) the study reported CPAK types within their cohorts. Exclusion criteria encompassed studies that employed modified radiological measurement techniques, utilized classification systems other than CPAK, or excluded certain patients based on coronal phenotype.

### Study selection and data extraction

To determine eligibility, two authors independently screened the titles and abstracts of all identified records. In cases where titles suggested relevance, full-text articles were assessed. Subsequently, two authors independently extracted data from the eligible studies. All discrepancies were resolved through discussion, with input from a third senior author, and final decisions were made by consensus. Following initial screening, the following data were extracted from relevant articles: study design, first author, publication year, country of origin, patient count, number of healthy and arthritic knees, sex, age, body mass index (BMI), LDFA, MPTA, mechanical hip-knee-ankle angle (mHKA), aHKA, JLO, and CPAK type distribution.

### Assessment of methodological quality

Two authors independently assessed the methodological quality of the included studies using the Hoy Risk of Bias Tool, which has been widely applied to evaluate prevalence studies of various health conditions with different designs [11]. This tool provides a summary score representing the risk of bias based on ten domains, each scored as 0 (absence of bias) or 1 (presence of bias). A summary score of 0 to 3 indicates a low risk of bias, 4 to 6 indicates a moderate risk of bias, and 7 to 9 indicates a high risk of bias. Any discrepancies in the quality assessment were resolved through consensus.

### Data analyses

A qualitative and quantitative synthesis of the results extracted from each included study was conducted. Continuous variables were expressed as means ± standard deviations (SD), while categorical variables were reported as absolute and relative frequencies. For geographic areas where multiple studies reported CPAK distribution, a meta-analysis of proportions was performed to estimate the overall prevalence of each CPAK type. Statistical analyses were conducted using STATA 12.0 (Stata Corp LLC, College Station, TX, USA). Differences between countries or regions were illustrated using a world map combined with a histogram. Chi-squared tests were conducted to evaluate the proportional differences between the most common CPAK types among all studies for healthy and arthritic knees, with *P*-values < 0.05 considered statistically significant.

## Results

### Included studies

**Fig. 2:**
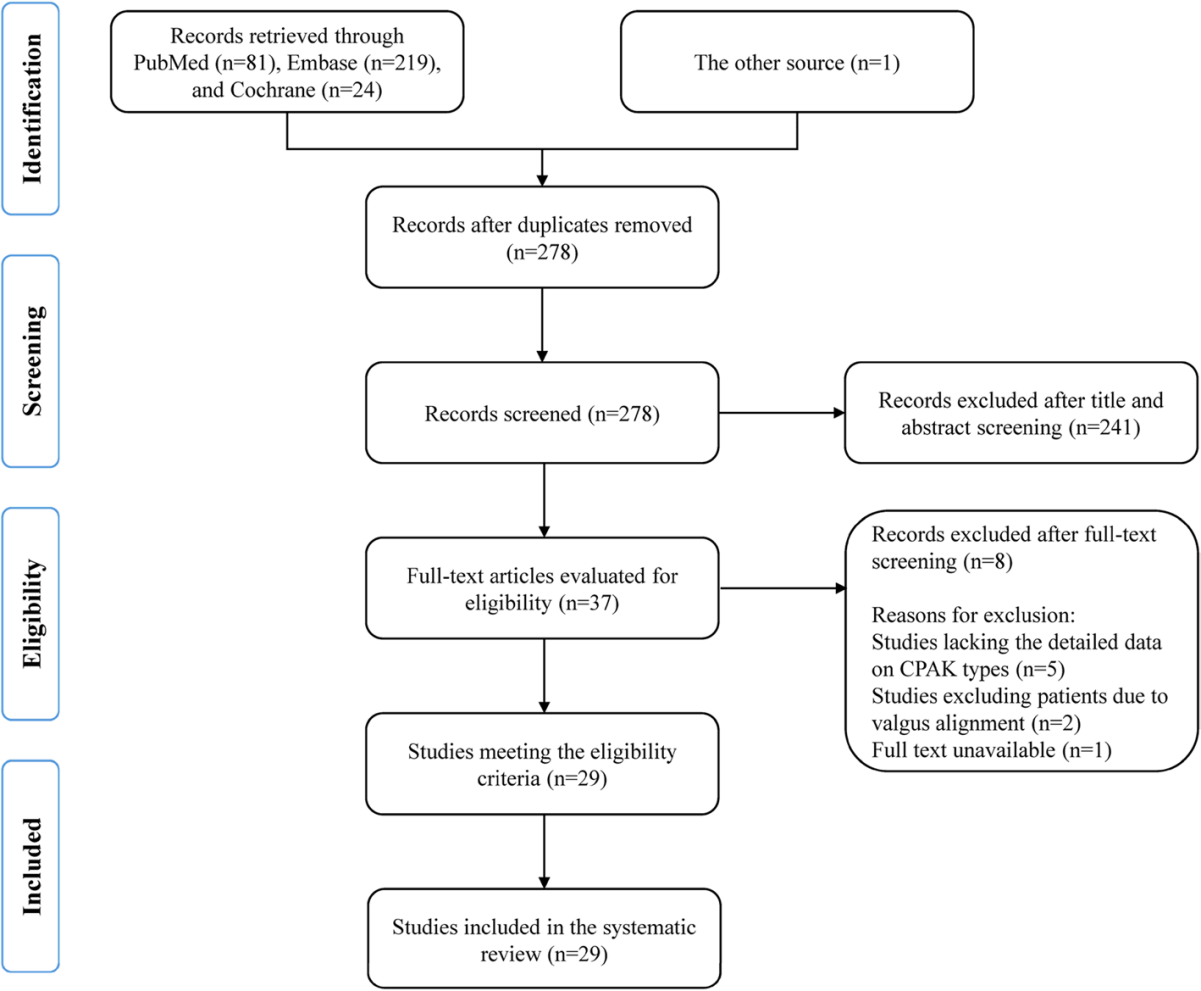
PRISMA search flow diagram

The literature search identified 325 potentially relevant records. After removing duplicates and conducting title and abstract screening, full-text assessments were performed against the inclusion criteria, resulting in the inclusion of 29 studies (28 retrospective and 1 prospective) [5–8, 12–36]. The PRISMA flowchart was used to illustrate the selection process (Fig. 2). The methodological quality of the included studies was assessed using the Hoy Risk of Bias Tool, and the quality of 28 studies was good; one study was considered to have a moderate risk of bias; no study was considered to have a high risk of bias (Table 1). The 29 included studies comprised 22,342 subjects with 27,660 knees. This included 4,082 healthy knees from 3,268 subjects from seven countries and 21,632 arthritic knees from 18,101 patients from 13 countries. One study included both healthy and arthritic knees without providing separate data [33]. Data on healthy people in both studies came from the same source [5, 27]. Baseline characteristics of the included studies are described in Table 2. The coronal plane angular measurements are shown in Table 3, and the distribution of CPAK among the included studies is presented in Table 4.

**Table 1:** Risk of bias assessment tool, adapted from the Risk of Bias Tool for Prevalence Studies developed by Hoy et al

Studies	Assessment Domain									
	1. Was the study’s target population a close representation of the national population in relation to relevant variables, e.g., age, sex, occupation?	2. Was the sampling frame a true or close representation of the target population?	3. Was some form of random selection used to select the sample, OR was a census undertaken?	4. Was the likelihood of non-response bias minimal?	5. Were data collected directly from the subjects (as opposed to a proxy)?	6. Was an acceptable case definition used in the study?	7. Was the study instrument that measured the parameter of interest shown to have reliability and validity (if necessary)?	8. Was the same mode of data collection used for all subjects?	9. Were the numerator(s) and denominator(s) for the parameter of interest appropriate?	10. Risk level (0–3, Low; 4–6, moderate; 7–9, high)
MacDessi et al. [27]	N	Y	Y	Y	Y	Y	Y	Y	Y	1, Low
Corban et al. [12]	N	Y	Y	Y	Y	Y	Y	Y	Y	1, Low
Tarassoli et al. [36]	N	Y	N	Y	Y	Y	Y	Y	Y	2, Low
Tarassoli et al. [35]	N	N	Y	Y	Y	Y	N	Y	Y	3, Low
Hazratwala et al. [18]	N	N	Y	Y	Y	Y	N	Y	Y	3, Low
Kim et al. [21]	N	N	N	Y	Y	Y	N	Y	Y	4, Moderate
Moore et al. [28]	N	Y	N	Y	Y	Y	Y	Y	Y	2, Low
Franceschetti et al. [14]	N	N	N	Y	Y	Y	Y	Y	Y	3, Low
Dragosloveanu et al. [13]	N	Y	Y	Y	Y	Y	Y	Y	Y	1, Low
Huber et al. [20]	N	Y	Y	Y	Y	Y	Y	Y	Y	1, Low
León-Muñoz et al. [23]	N	Y	Y	Y	Y	Y	Y	Y	Y	1, Low
Sappey-Marinier et al. [32]	N	Y	Y	Y	Y	Y	Y	Y	Y	1, Low
Loddo et al. [26]	N	Y	Y	Y	Y	Y	Y	Y	Y	1, Low
Pangaud et al. [31]	N	N	N	Y	Y	Y	Y	Y	Y	3, Low
Şenel et al. [33]	N	Y	Y	Y	Y	Y	Y	Y	Y	1, Low
Steele et al. [34]	N	Y	Y	Y	Y	Y	Y	Y	Y	1, Low
Grant et al. [16]	N	Y	Y	Y	Y	Y	N	Y	Y	1, Low
Morrisey et al. [29]	N	N	N	Y	Y	Y	Y	Y	Y	3, Low
Hsu et al. [19]	N	Y	Y	Y	Y	Y	Y	Y	Y	1, Low
Li et al. [24]	N	Y	Y	Y	Y	Y	Y	Y	Y	1, Low
Liu et al. [25]	N	N	N	Y	Y	Y	Y	Y	Y	3, Low
Gao et al. [15]	N	Y	Y	Y	Y	Y	Y	Y	Y	1, Low
Toyooka et al. [6]	N	Y	Y	Y	Y	Y	Y	Y	Y	1, Low
Nomoto et al. [7]	N	Y	N	Y	Y	Y	Y	Y	Y	2, Low
Harada et al. [17]	N	Y	Y	Y	Y	Y	Y	Y	Y	1, Low
Konishi et al. [22]	N	Y	Y	Y	Y	Y	Y	Y	Y	1, Low
Mulpur et al. [30]	N	Y	Y	Y	Y	Y	Y	Y	Y	1, Low
Yang et al. [37]	N	Y	Y	Y	Y	Y	Y	Y	Y	1, Low
Coetzee et al. [8]	N	Y	Y	Y	Y	Y	Y	Y	Y	1, Low

N, no, means the absence of bias; Y, yes, means the present of bias

**Table 2:** Baseline characteristics of included studies

**Studies**	Country/ Region	Number of knees	Number of subjects	Healthy or arthritic	Mean age, years (SD/range)	Sex (*n*, %)		BMI (SD/range)	Study design
						Men	Women		
MacDessi et al. [5]	Belgium	500	250	Healthy	22 (20–27)	125 (50.0%)	125 (50.0%)	22.0 (2.9)	Retrospective cross-sectional
	Australia	500	500	Arthritic	66 (44–88)	190 (38.0%)	310 (62.0%)	NA	
Corban et al. [12]	Australia	700	643	Arthritic	68.2 (7.9)	305 (48.6%)	395 (61.4%)	29.7 (5.7)	Retrospective cohort
Tarassoli et al. [35]	Australia	88	76	Arthritic	68 (42–87)	41 (53.9%)	35 (46.1%)	NA	Retrospective
Tarassoli et al. [34]	Australia	465	394	Arthritic	69.4 (46–89)	179 (45.4%)	215 (54.6%)	29.59	Retrospective
Hazratwala et al. [18]	Australia	165	140	Arthritic	65.1 (8.3)	63 (45.0%)	77 (55.0%)	NA	Retrospective case series review
Kim et al. [21]	Australia	1124	1124	Arthritic	NA	NA	NA	NA	Retrospective review
Moore et al. [27]	Belgium	500	250	Healthy	22 (20–27)	125 (50.0%)	125 (50.0%)	22.0 (2.9)	retrospective case–control
	Australia	710	355	Arthritic	70.2 (7.6)	162 (45.6%)	193 (54.4%)	29.7 (4.8)	
Franceschetti et al. [14]	Italy	180	180	Arthritic	NA	73 (40.6%)	107 (59.4%)	NA	Retrospective cohort
Dragosloveanu et al. [13]	Romania	500	500	Healthy	36.0 (14.2)	330 (66.0%)	170 (34.0%)	26.5 (4.2)	Observational cross-sectional
	Romania	500	500	Arthritic	68.0 (7.2)	125 (25.4%)	375 (74.6%)	30.8 (4.0)	
Huber et al. [20]	Austria	8739	7456	Arthritic	69.0 (9.3)	2502 (33.6%)	4954 (66.4%)	30.3 (5.6)	Retrospective
León-Muñoz et al. [23]	Spain	501	447	Arthritic	69.9 (6.3)	159 (35.6%)	288 (64.4%)	29.8 (3.9)	Retrospective cross-sectional
Sappey-Marinier et al. [31]	France	1078	936	Arthritic	71.3 (8.0)	780 (83.3%)	156 (16.7%)	29.2 (5.1)	Retrospective cohort
Loddo et al. [26]	France	1240	1240	Healthy	58.9 (14.5)	658 (53.1%)	582 (46.9%)	25.9 (6.3)	Retrospective diagnostic
Pangaud et al. [30]	France	178	178	Arthritic	70.3 (7.1)	NA	NA	29.6 (4.3)	Retrospective cohort
Şenel et al. [32]	Turkey	414	207	Healthy	32.9 (8.4)	109 (52.7%)	98 (47.3%)	NA	Retrospective cross-sectional
	Turkey	408	296	Arthritic	54.5 (7.9)	141 (47.6%)	155 (52.4%)	NA	
Steele et al. [33]	USA	1946	973	Mixed	61 (9.1)	477 (49.0%)	496 (51.0%)	29.3 (19–44)	Retrospective cohort
Grant et al. [16]	USA	1166	1166	Arthritic	NA	520 (44.6%)	646 (55.4%)	NA	Retrospective
Morrisey et al. [28]	USA	335	335	Arthritic	69.2 (8.1)	135 (40.3%)	200 (59.7%)	NA	Retrospective
Hsu et al. [19]	China	214	214	Healthy	41 (18.6)	111 (51.9%)	103 (48.1%)	NA	Retrospective cross-sectional
Li et al. [24]	China	944	479	Arthritic	67.6 (6.4)	101 (21.1%)	378 (78.9%)	26.8 (3.4)	Retrospective
Liu et al. [25]	China	434	434	Arthritic	66.4 (9.3)	93 (21.4%)	341 (78.6%)	25.5 (3.7)	Retrospective
Gao et al. [15]	China	214	107	Healthy	48.8 (14.4)	41 (38.3%)	66 (61.7%)	NA	Retrospective
	China	477	246	Arthritic	65.3 (7.3)	65 (26.4%)	181 (73.6%)	NA	
Toyooka et al. [6]	Japan	500	343	Arthritic	75.1 (8.0)	95 (19.0%)	405 (81.0%)	26.2 (4.0)	Retrospective
Nomoto et al. [7]	Japan	248	248	Arthritic	NA	79 (31.9%)	169 (68.1%)	NA	Retrospective cohort
Harada et al. [17]	Japan	300	300	Arthritic	NA	150 (50.0%)	150 (50.0%)	NA	Retrospective cross-sectional
Konishi et al. [22]	Japan	284	231	Arthritic	74.0 (8.0)	33 (14.3%)	198 (85.7%)	26.7 (4.4)	Retrospective cohort
Mulpur et al. [29]	India	500	250	Healthy	26.8 (4.5)	109 (43.6%)	141 (56.4%)	25.8 (4.8)	Prospective cross-sectional
	India	500	250	Arthritic	62.3 (8.2)	76 (30.4%)	174 (69.6%)	28.2 (4.0)	
Yang et al. [36]	Korea Korea	500	500	Healthy	23.8 (8.2)	416 (82.2%)	84 (16.8%)	24.8 (8.9)	Retrospective cross-sectional
		500	500	Arthritic	75.0 (4.0)	419 (83.8%)	81 (16.2%)	26.1 (4.0)	
Coetzee et al. [8]	South Africa	608	344	Arthritic	68.4 (9.2)	76 (22.1%)	268 (77.9%)	NA	Retrospective cross-sectional

NA, not available. BMI, body mass index

**Table 3:** Angular measurements among included studies

Studies	Country/Region	The number of knees (healthy/arthritic/mixed)	Mean LDFA (SD)	Mean MPTA (SD)	Mean mHKA (SD)	Mean aHKA (SD)	Mean JLO (SD)
MacDessi et al. [5]	Belgium	500 (healthy)	87.9 (1.7)	87.0 (2.1)	− 1.3 (2.3)	− 0.9 (2.5)	175.0 (2.5)
	Australia	500 (arthritic)	88.1 (2.1)	87.3 (2.1)	− 2.9 (7.4)	− 0.8 (2.8)	175.5 (3.1)
Corban et al. [12]	Australia	700 (arthritic)	87.4 (4.0)	87.3 (2.8)	NA	− 0.1 (4.0)	174.7 (3.3)
Tarassoli et al. [35]	Australia	88 (arthritic)	NA	NA	− 3.6 (6.7)	− 0.3 (3.9)	NA
Tarassoli et al. [34]	Australia	465 (arthritic)	87.7 (2.3)	87.5 (2.8)	NA	− 0.2 (3.9)	175.1 (3.3)
Hazratwala et al. [18]	Australia	165 (arthritic)	87.5 (2.9)	87.1 (2.6)	− 3.8 (6.2)	NA	NA
Kim et al. [21]	Belgium	1,124 (arthritic)	NA	NA	NA	NA	NA
Moore et al. [27]	Australia	500 (healthy)	87.9 (1.7)	87.0 (2.1)	− 1.3 (2.3)	− 0.9 (2.5)	175.0 (2.5)
	Australia	710 (arthritic)	87.8 (2.6)	87.3 (3.0)	− 3.8 (6.6)	− 0.5 (4.6)	175.1 (3.3)
Franceschetti et al. [14]	Italy	180 (arthritic)	NA	NA	NA	NA	NA
Dragosloveanu et al. [13]	Romania	500 (healthy)	87.3 (2.2)	87.1 (2.3)	NA	− 0.2 (3.1)	174.3 (3.2)
	Romania	500 (arthritic)	88.8 (3.2)	86.2 (3.4)	NA	− 2.6 (5.2)	175.0 (4.1)
Huber et al. [20]	Austria	8,739 (arthritic)	87.3 (2.8)	87.2 (3.2)	− 2.7 (7.6)	− 0.1 (4.8)	174.5 (3.6)
León-Muñoz et al. [23]	Spain	501 (arthritic)	89.8 (2.8)	86.4 (2.8)	− 7.7 (6.2)	− 3.4 (4.3)	176.2 (3.6)
Sappey-Marinier et al. [31]	France	1,078 (arthritic)	88.5 (2.6)	86.8 (3.1)	NA	NA	NA
Loddo et al. [26]	France	1,240 (healthy)	86.9 (2.5)	85.4 (2.4)	− 1.4 (4.4)	NA	NA
Pangaud et al. [30]	France	178 (arthritic)	88.9 (2.8)	85.9 (3.9)	− 4.6 (7.5)	NA	NA
Şenel et al. [32]	Turkey	414 (healthy)	88.0 (2.3)	87.2 (1.9)	NA	0.3 (2.5)	175.2 (3.5)
	Turkey	408 (arthritic)	88.0 (2.9)	86.6 (2.6)	NA	− 1.4 (3.9)	174.6 (3.7)
Steele et al. [33]	USA	1,946 (mixed)	87.7 (2.1)	87.6 (2.5)	− 1.2 (3.8)	− 0.4 (3.3)	175.3 (3.2)
Grant et al. [16]	USA	1,166 (arthritic)	NA	NA	NA	NA	NA
Morrisey et al. [28]	USA	335 (arthritic)	NA	NA	NA	NA	NA
Hsu et al. [19]	China	214 (healthy)	87.3 (2.4)	85.8 (2.2)	− 1.2 (3.1)	− 1.5 (3.2)	173.1 (3.3)
Li et al. [24]	China	944 (arthritic)	88.7 (3.6)	85.7 (3.5)	NA	− 3.0 (5.7)	174.5 (4.3)
Liu et al. [25]	China	434 (arthritic)	88.7 (3.2)	84.7 (4.4)	− 7.9 (7.2)	− 4.0 (6.2)	173.4 (4.5)
Gao et al. [15]	China	214 (healthy)	86.2 (2.4)	86.3 (2.7)	− 2.3 (3.6)	0.2 (3.7)	172.5 (3.6)
	China	477 (arthritic)	88.6 (3.6)	85.0 (3.8)	− 6.4 (6.7)	− 3.6 (5.8)	173.6 (4.6)
Toyooka et al. [6]	Japan	500 (arthritic)	88.0 (2.9)	84.4 (3.3)	− 12.8 (4.7)	− 3.5 (4.8)	172.4 (3.8)
Nomoto et al. [7]	Japan	248 (arthritic)	87.4 (3.2)	83.7 (2.8)	− 4.8 (3.9)	− 3.6 (3.8)	171.1 (4.6)
Harada et al. [17]	Japan	300 (arthritic)	NA	NA	NA	NA	NA
Konishi et al. [22]	Japan	284 (arthritic)	NA	NA	NA	NA	NA
Mulpur et al. [29]	India	500 (healthy)	88.9 (3.0)	87.1 (2.8)	NA	− 1.7 (3.5)	176.0 (4.5)
	India	500 (arthritic)	90.2 (3.6)	83.4 (3.4)	NA	− 6.9 (5.0)	173.5 (5.0)
Yang et al. [36]	Korea	500 (healthy)	87.9 (2.3)	87.1 (2.6)	− 1.0 (2.9)	− 0.8 (3.0)	175.0 (3.0)
	Korea	500 (arthritic)	89.3 (3.0)	84.7 (3.0)	− 10.3 (4.6)	− 4.6 (3.8)	174.0 (3.5)
Coetzee et al. [8]	South Africa	608 (arthritic)	87.2 (3.0)	88.2 (2.8)	− 1.7 (8.8)	1.0 (4.8)	175.2 (3.4)

LDFA, lateral distal femoral angle; MPTA, medial proximal tibial angle; mHKA, mechanical hip-knee-ankle angle; aHKA, arithmetic hip-knee-ankle angle; JLO, joint line obliquity. NA, not available

**Table 4:** Coronal Plane Alignment of the Knee (CPAK) Classification distributions among included studies

Studies	Country/Region	The number of knees (healthy/arthritic/mixed)	Type I (%)	Type II (%)	Type III (%)	Type IV (%)	Type V (%)	Type VI (%)	Type VII (%)	Type VIII (%)	Type IX (%)
MacDessi et al. [5]	Belgium	500 (healthy)	132 (26.4)	196 (39.2)	49 (9.8)	27 (5.4)	77 (15.4)	17 (3.4)	1 (0.2)	0 (0.0)	1 (0.2)
	Australia	500 (arthritic)	97 (19.4)	161 (32.2)	77 (15.4)	49 (9.8)	73 (14.6)	37 (7.4)	3 (0.6)	8 (1.6)	2 (0.4)
Corban et al. [12]	Australia	700 (arthritic)	177 (25.3)	204 (29.1)	153 (21.9)	44 (6.3)	69 (9.9)	48 (6.9)	1 (0.1)	2 (0.3)	2 (0.1)
Tarassoli et al. [35]	Australia	88 (arthritic)	29 (33.0)	24 (27.3)	14 (15.9)	3 (3.4)	5 (5.7)	11 (12.5)	0 (0.0)	1 (1.1)	1 (1.1)
Tarassoli et al. [34]	Australia	465 (arthritic)	112 (24.1)	149 (32.0)	80 (17.2)	36 (7.7)	40 (8.6)	42 (9.0)	0 (0.0)	3 (0.7)	3 (0.7)
Hazratwala et al. [18]	Australia	165 (arthritic)	34 (20.4)	63 (37.9)	27 (16.3)	11 (6.6)	21 (12.7)	8 (4.8)	2 (1.2)	0 (0.0)	0 (0.0)
Kim et al. [21]	Australia	1,124 (arthritic)	355 (31.6)	245 (21.8)	211 (18.8)	83 (7.4)	70 (6.2)	114 (10.1)	11 (1.0)	4 (0.4)	31 (2.8)
Moore et al. [27]	Belgium	500 (healthy)	132 (26.4)	196 (39.2)	49 (9.8)	27 (5.4)	77 (15.4)	17 (3.4)	1 (0.2)	0 (0.0)	1 (0.2)
	Australia	710 (arthritic)	208 (29.3)	192 (27.0)	130 (18.3)	62 (8.7)	59 (8.3)	50 (7.0)	1 (0.1)	2 (0.3)	6 (0.9)
Franceschetti et al. [14]	Italy	180 (arthritic)	43 (23.9)	41 (22.8)	24 (13.3)	22 (12.2)	23 (12.8)	17 (9.4)	5 (2.8)	3 (1.7)	2 (1.1)
Dragosloveanu et al. [13]	Romania	500 (healthy)	112 (22.4)	195 (39.0)	93 (18.6)	25 (5.0)	48 (9.6)	22 (4.4)	3 (0.6)	1 (0.2)	1 (0.2)
	Romania	500 (arthritic)	212 (42.4)	84 (16.8)	49 (9.8)	81 (16.2)	37 (7.4)	23 (4.6)	6 (1.2)	5 (1.0)	3 (0.6)
Huber et al. [20]	Austria	8,739 (arthritic)	2,454 (28.1)	2,383 (27.3)	1,830 (20.9)	539 (6.2)	658 (7.5)	754 (8.6)	30 (0.3)	29 (0.3)	62 (0.7)
León-Muñoz et al. [23]	Spain	501 (arthritic)	154 (30.7)	105 (21.0)	25 (5.0)	130 (25.9)	56 (11.2)	14 (2.8)	12 (2.4)	3 (0.6)	2 (0.4)
Sappey-Marinier et al. [31]	France	1,078 (arthritic)	360 (33.4)	210 (19.5)	115 (10.6)	110 (10.2)	204 (18.9)	68 (6.3)	4 (0.4)	6 (0.6)	1 (0.1)
Loddo et al. [26]	France	1,240 (healthy)	382 (30.8)	571 (46.0)	159 (12.8)	51 (4.1)	58 (4.7)	15 (1.2)	2 (0.2)	1 (0.1)	1 (0.1)
Pangaud et al. [30]	France	178 (arthritic)	80 (44.9)	41 (23.1)	18 (10.1)	16 (8.9)	8 (4.5)	7 (3.9)	2 (1.1)	0 (0.0)	6 (3.5)
Şenel et al. [32]	Turkey	414 (healthy)	60 (14.5)	172 (41.5)	61 (14.7)	21 (5.1)	49 (11.8)	45 (10.9)	0 (0.0)	2 (0.5)	4 (1.0)
	Turkey	408 (arthritic)	115 (28.2)	129 (31.6)	55 (13.5)	42 (10.3)	50 (12.3)	10 (2.5)	4 (1.0)	0 (0.0)	3 (0.7)
Steele et al. [33]	USA	1,946 (mixed)	193 (19.8)	335 (34.5)	170 (17.5)	62 (6.4)	122 (12.6)	80 (8.2)	4 (0.4)	2 (0.2)	5 (0.5)
Grant et al. [16]	USA	1,166 (arthritic)	428 (36.7)	193 (16.6)	81 (6.9)	192 (16.5)	106 (9.1)	40 (3.4)	81 (6.9)	33 (2.8)	12 (1.0)
Morrisey et al. [28]	USA	335 (arthritic)	76 (22.7)	158 (47.2)	31 (9.2)	14 (4.2)	46 (13.6)	11 (3.3)	0 (0.0)	0 (0.0)	0 (0.0)
Hsu et al. [19]	China	214 (healthy)	78 (36.4)	84 (39.3)	29 (13.6)	12 (5.6)	10 (4.7)	1 (0.5)	0 (0.0)	0 (0.0)	0 (0.0)
Li et al. [24]	China	944 (arthritic)	359 (38.0)	189 (20)	101 (10.7)	161 (17.1)	72 (7.6)	45 (4.8)	10 (1.1)	5 (0.5)	2 (0.2)
Liu et al. [25]	China	434 (arthritic)	234 (53.9)	74 (17.1)	40 (9.2)	55 (12.7)	10 (2.3)	13 (3.0)	4 (0.9)	1 (0.2)	3 (0.7)
Gao et al. [15]	China	214 (healthy)	49 (22.9)	96 (44.9)	51 (23.8)	3 (1.4)	12 (5.6)	3 (1.4)	0 (0.0)	0 (0.0)	0 (0.0)
	China	477 (arthritic)	208 (43.6)	103 (21.6)	50 (10.5)	55 (11.5)	36 (7.5)	18 (3.8)	5 (1.1)	0 (0.0)	2 (0.4)
Toyooka et al. [6]	Japan	500 (arthritic)	269 (53.8)	127 (25.4)	41 (8.2)	36 (7.2)	22 (4.4)	5 (1.0)	0 (0.0)	0 (0.0)	0 (0.0)
Nomoto et al. [7]	Japan	248 (arthritic)	161 (65.0)	58 (23.3)	17 (6.7)	4 (1.7)	4 (1.7)	0 (0.0)	4 (1.7)	0 (0.0)	0 (0.0)
Harada et al. [17]	Japan	300 (arthritic)	123 (41.0)	121 (40.3)	33 (11.0)	9 (3.0)	11 (3.7)	2 (0.7)	0 (0.0)	1 (0.3)	0 (0.0)
Konishi et al. [22]	Japan	284 (arthritic)	155 (54.6)	38 (13.4)	25 (8.8)	45 (15.8)	5 (1.8)	7 (2.5)	2 (0.7)	2 (0.7)	5 (1.8)
Mulpur et al. [29]	India	500 (healthy)	106 (21.2)	128 (25.6)	30 (6.0)	84 (16.8)	98 (19.6)	29 (5.8)	12 (2.4)	9 (1.8)	4 (0.8)
	India	500 (arthritic)	294 (58.8)	69 (13.8)	7 (1.4)	91 (18.2)	17 (3.4)	5 (1.0)	14 (2.8)	3 (0.6)	0 (0.0)
Yang et al. [36]	Korea	500 (healthy)	143 (28.6)	191 (38.2)	82 (16.4)	20 (4.0)	38 (7.6)	21 (4.2)	0 (0.0)	2 (0.4)	3 (0.6)
	Korea	500 (arthritic)	269 (53.8)	88 (17.6)	8 (1.6)	87 (17.4)	36 (7.2)	3 (0.6)	8 (1.6)	1 (0.2)	0 (0.0)
Coetzee et al. [8]	South Africa	608 (arthritic)	94 (15.5)	155 (25.5)	174 (28.6)	45 (7.4)	52 (8.6)	80 (13.2)	3 (0.5)	1 (0.2)	4 (0.7)

### Geographic differences

#### North America

North America was represented in three studies [16, 28, 33]. Steele et al. [33] examined the distribution of CPAK across a mixed cohort of 1,946 healthy and arthritic knees in 973 individuals from the Osteoarthritis Initiative (OAI). However, in this study, the CPAK types were not stratified by healthy and arthritic knees. Grant et al. [16] and Morrisey et al. [28] evaluated 1,501 arthritic knees from 1,501 individuals undergoing TKA in the United States. By synthesizing the data from these two studies, the pooled estimated prevalence of each CPAK type in arthritic knees in the USA was calculated. The most common type was CPAK type I (32.8%, 95% CI: 30.5% to 35.2%), followed by type II (20.8%, 95% CI: 18.8% to 22.7%) and type IV (10.4%, 95% CI: 8.9% to 11.9%).

#### Europe

Europe was represented in nine studies [5, 7, 13, 14, 20, 23, 26, 30, 31]. Four of these studies analyzed the distribution of CPAK types in 2,654 healthy knees among 2,197 individuals. Seven studies examined the distribution of CPAK types in 11,584 arthritic knees among 9,993 individuals. The pooled estimated prevalence of each CPAK type in healthy and arthritic knees in Europe was calculated by synthesizing the data from these studies. For healthy knees, CPAK type II was the most common (41.7%, 95% CI: 37.9% to 45.5%), followed by type I (23.6%, 95% CI: 16.2% to 30.9%) and type III (13.8%, 95% CI: 10.6% to 17.1%). For arthritic knees, CPAK type I was the most common (32.8%, 95% CI: 28.4% to 37.1%), followed by type II (23.1%, 95% CI: 19.0% to 27.2%) and type IV (12.7%, 95% CI: 8.4% to 17.1%).

#### Asia

Asia was represented in ten studies [6, 7, 15, 17, 19, 22, 24, 25, 29, 36]. Four of these studies examined the distribution of CPAK types in 1,428 healthy knees from 1,321 individuals. Nine studies looked at the distribution in 4,187 arthritic knees from 3,031 individuals. By synthesizing data from these studies, the pooled estimated prevalence of each CPAK type in both healthy and arthritic knees in Asia was calculated. In healthy knees, CPAK type II was the most common (36.7%, 95% CI: 28.1% to 45.4%), followed by type I (27.0%, 95% CI: 20.9% to 33.0%) and type III (14.7%, 95% CI: 7.2% to 22.2%). For arthritic knees, CPAK type I was predominant (51.3%, 95% CI: 45.3% to 57.3%), followed by type II (21.1%, 95% CI: 17.0% to 25.3%) and type IV (11.5%, 95% CI: 6.9% to 16.1%).

#### Australia

Australia was represented in seven studies that examined the distribution of CPAK types in 3,752 arthritic knees from 3,232 individuals [5, 12, 18, 21, 27, 34, 35]. The pooled estimated prevalence of each CPAK type in arthritic knees in Australia was determined by synthesizing the data from these studies. For arthritic knees, CPAK type II was the most common (29.3%, 95% CI: 25.3% to 33.2%), followed by type I (25.9%, 95% CI: 22.1% to 29.7%) and type III (18.1%, 95% CI: 16.5% to 19.8%).

#### Africa

Africa was represented in only one study. Coetzee et al. [8] examined the distribution of CPAK types in 608 arthritic knees from 344 individuals. In their study, CPAK type III was the most common (28.6%), followed by type II (25.5%) and type I (15.5%).

#### Geographic differences

**Fig. 3:**
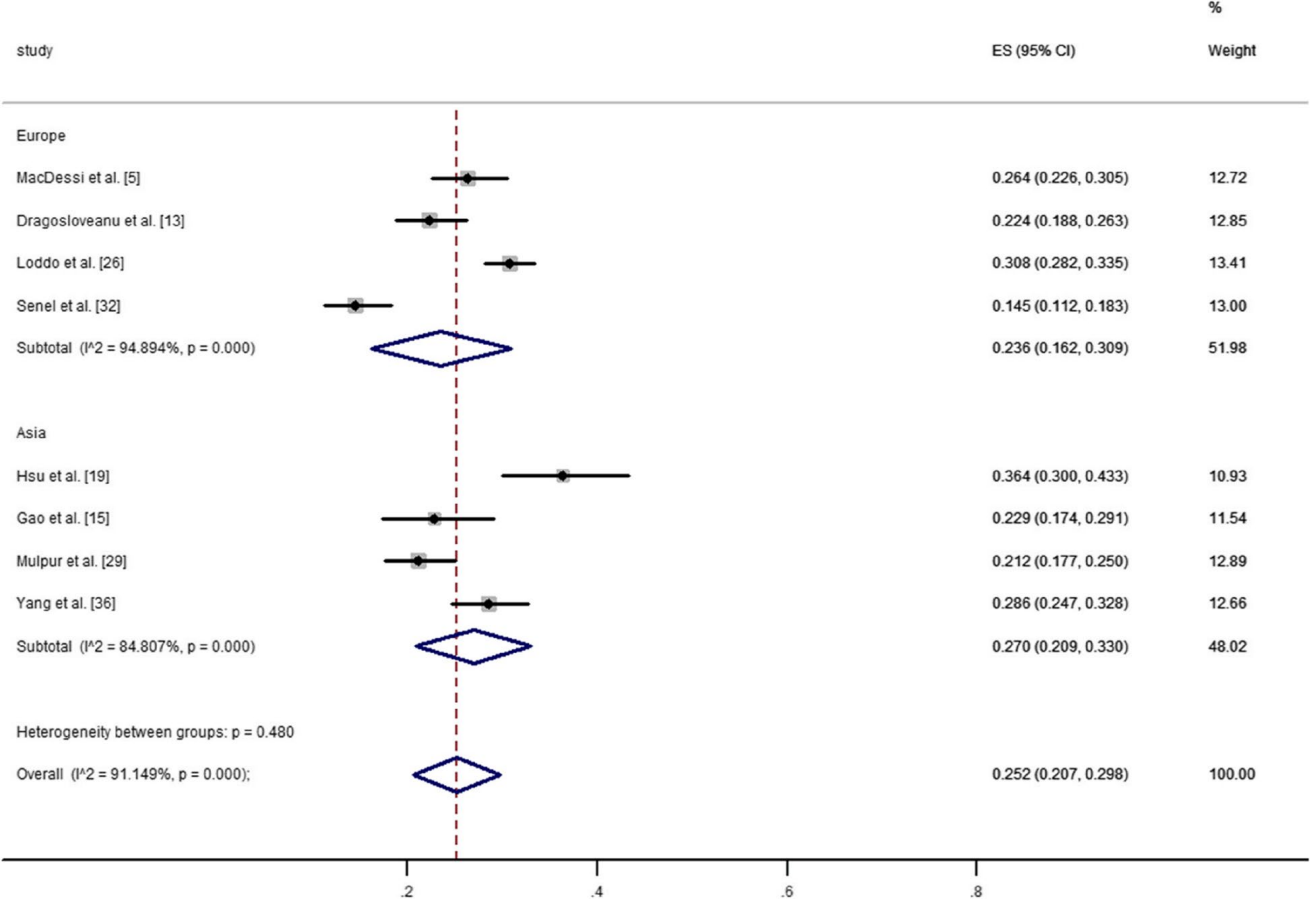
The prevalence difference of Coronal Plane Alignment of the Knee (CPAK) types I among different geographical regions in healthy knees

**Fig. 4:**
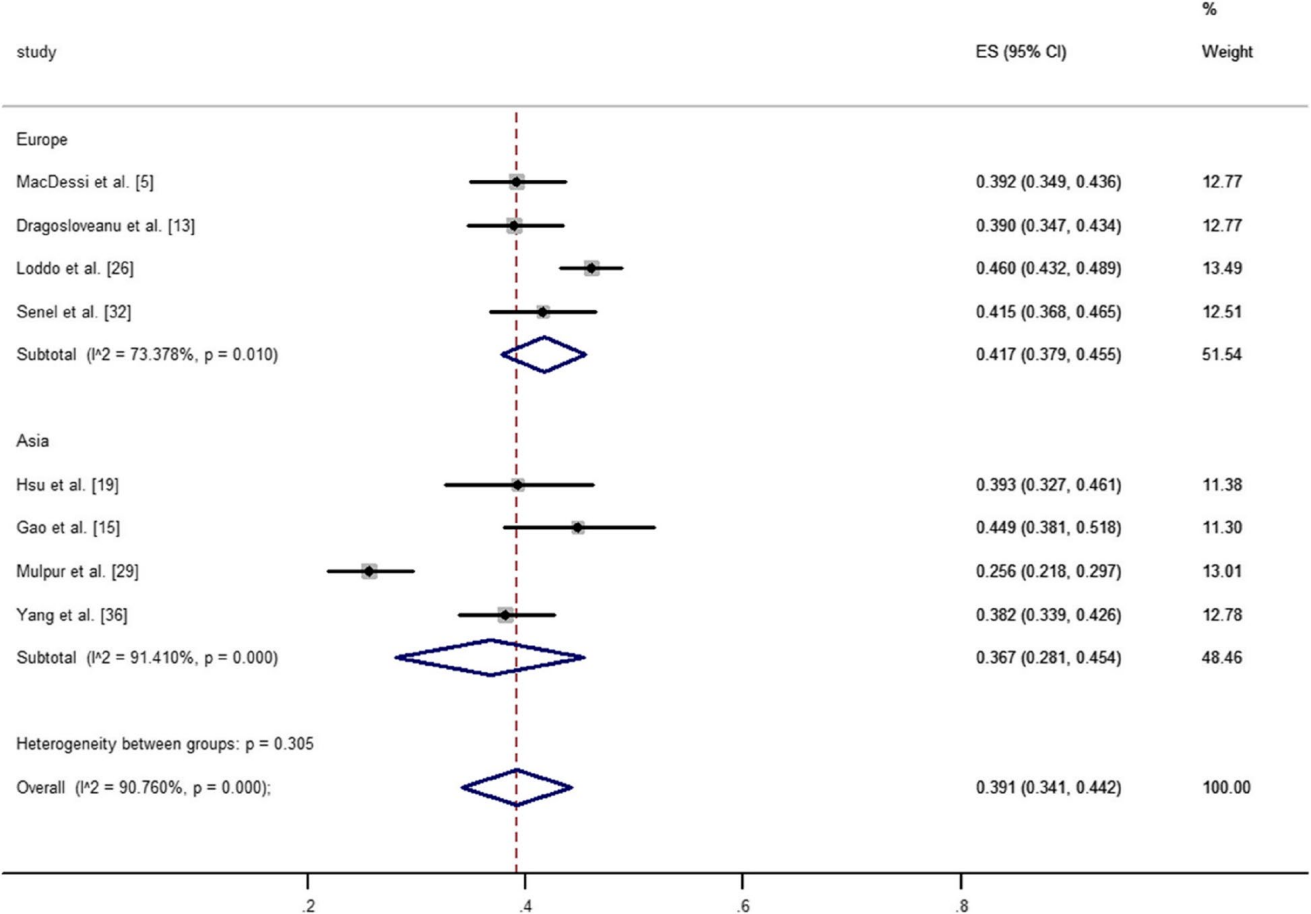
The prevalence difference of Coronal Plane Alignment of the Knee (CPAK) types II among different geographical regions in healthy knees

**Fig. 5:**
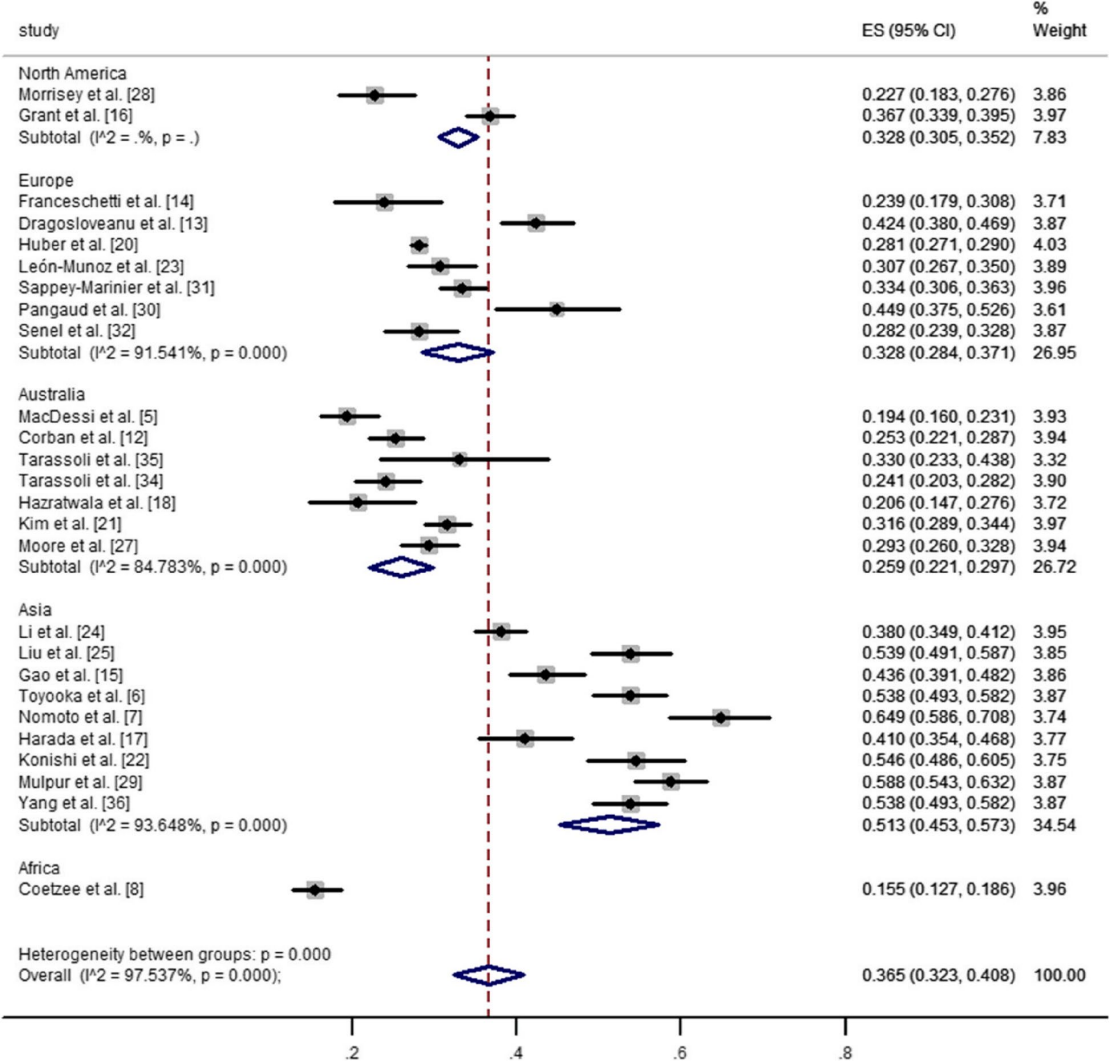
The prevalence difference of Coronal Plane Alignment of the Knee (CPAK) types I among different geographical regions in arthritic knees

**Fig. 6:**
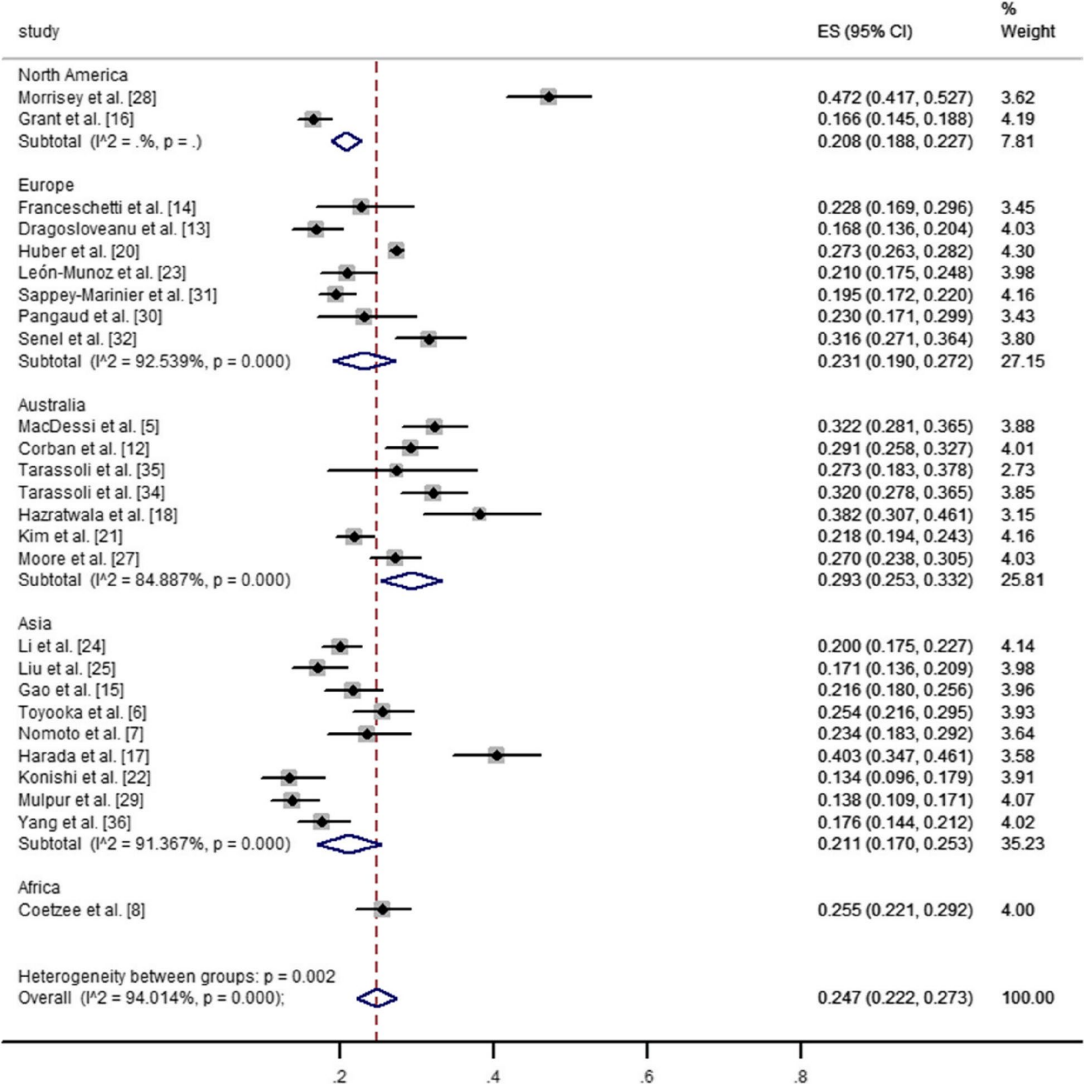
The prevalence difference of Coronal Plane Alignment of the Knee (CPAK) types II among different geographical regions in arthritic knees

The prevalence of CPAK types I and II among different geographical regions in healthy and arthritic knees was illustrated in Figs. 3, 4, 5 and 6. The pooled estimated prevalence of each CPAK type in healthy or arthritic knees was determined by synthesizing the data from included studies. Among healthy knees, no significant differences were observed in the prevalence of CPAK type I (23.6% VS. 27.0%, *P* = 0.48) and CPAK type II (41.7% vs. 36.7%, *P* = 0.305) between Europe and Asia (Figs. 3 and 4). In arthritic knees, significant differences were found in the prevalence of CPAK type I (32.8% vs. 32.8% vs. 25.9% vs. 51.3% vs. 15.5%, *P* < 0.001) and CPAK type II (20.8% vs. 23.1% vs. 29.3% vs. 21.1% vs. 25.5%, *P* = 0.002) across North America, Europe, Australia, Asia and Africa (Figs. 5 and 6). Interestingly, although significant differences were found in the prevalence of CPAK type I between Europe and Asia (32.8% vs. 51.3%, *P* < 0.001), no significant differences were observed in the prevalence of CPAK type II (23.1% VS. 21.1%, *P* = 0.508) between Europe and Asia.

## Discussion

This systematic review summarizes the current literature on the distribution of CPAK types across 14 countries in five continents. There is significant geographic variation in the distribution of CPAK types among arthritic populations, while no significant differences were observed among healthy populations. Additionally, the findings also indicate differences in the prevalence of CPAK types between healthy and arthritic populations within the same region.

Historically, the neutral coronal alignment of the lower limbs has generally been regarded as “normal alignment” [37, 38]. In 2012, Bellemans et al. [39] first introduced the concept of constitutional varus, demonstrating that a substantial proportion of the normal population in Belgium exhibits limb alignment outside the neutral mechanical alignment. Furthermore, Hovinga and Lerner [40] reported significant differences in alignment parameters, with Japanese subjects displaying more frequent and pronounced varus alignment compared to Caucasians. Similarly, Hsu et al. [19] and Nayak et al. [41] evaluated arthritic patients in Asia and found that 33.6% to 65.8% of the limbs were in varus (HKA < 177°). These findings highlight the importance of reconsidering the concept of normal coronal alignment of the lower limbs in different populations worldwide.

Traditionally, achieving neutral alignment has been a common objective for orthopedic surgeons performing TKAs [42, 43]. However, dissatisfaction following TKA is a widely recognized issue [2, 44]. The concept of kinematically aligned (KA), first introduced by Howell et al. [45], aims to restore the pre-arthritic HKA and JLO. However, the KA approach primarily focuses on the coronal position, potentially neglecting overall limb alignment, which could expose patients with significant deformities to the risk of early failure related to alignment [46, 47]. With the advent of advanced technological aids, the number of alignment types and techniques available to achieve a well-balanced TKA has increased significantly. Among the alignment types are adjusted mechanical alignment (aMA), restricted kinematic alignment (rKA), and inverse kinematic alignment (iKA) [48–51]. Moreover, instrumentation and techniques for TKA have evolved from conventional manual tools to a wide range of technologies, including calibrated guides for accurate bone cuts and alignment, smart tools, dynamic intraoperative sensors for soft tissue balancing, patient-specific guides, computer navigation, and robotics [52]. However, no matter what alignment or technique is chosen, it is crucial to understand the patient's native alignment.

Previous studies have demonstrated that categorizing coronal alignment into varus, valgus, or neutral is insufficient, as it captures only a static moment without accounting for joint line orientation [5, 53–55]. Consequently, the concept of knee phenotype was introduced, which encompasses the observable characteristics of the knee, including morphology, alignment, and laxity, providing a comprehensive characterization. In 2018, Lin et al. [56] proposed a classification system comprising 27 possible phenotypes, but only five were deemed clinically relevant. Hirschmann et al. [55] later introduced a novel classification system based on the functional knee phenotype concept, which includes 125 possible phenotypes, with 43 considered clinically relevant. While this method covers a broad spectrum, it presents numerous typing options and a complex process. Recently, the CPAK classification has garnered significant attention [5]. Despite being introduced less than four years ago, the CPAK system has exhibited excellent interrater reliability, relies solely on long-leg radiographs, and has been adopted worldwide.

Despite CPAK Type V (neutral anatomical HKA and neutral JLO) being the target for MA, MacDessi et al. [5] found that only 15% of the studied populations fell within the classification boundaries for this type. In our current study, we similarly observed that less than 20% of the population aligned with CPAK Type V. In regions with a particularly low prevalence of CPAK Type V, such as Japan (4.7%), the universal application of the MA approach may not be optimal. CPAK Type II knees (neutral aHKA and apex distal JLO) constituted nearly 40% (36.7% to 41.7%) of knees in the normal population and over 20% (20.8% to 29.3%) in the arthritic population. This CPAK type forms the basis for the anatomical axis (AA) method described by Hungerford et al. [56]. Although this technique aimed to align the joint line based on mean population values of 3° femoral valgus and 3° tibial varus, precise replication of these resection targets using conventional instrumentation proved challenging, leading to its eventual abandonment. CPAK Type I knees (varus aHKA with apex distal JLO) accounted for approximately 25% (23.6% to 27.0%) of knees in the normal population and more than 50% (25.9% to 51.3%) in the arthritic population. When MA is employed for such cases, substantial interventions are typically required to restore balance, often involving either varus resections or extensive medial collateral ligament (MCL) release.

Notably, our study revealed that the knee phenotype in South Africa differed significantly from those observed in other regions. CPAK Type III, characterized by constitutional valgus aHKA with an apex distal and neutral JLO, comprised nearly 30% of cases in South Africa, a proportion not previously reported in prior literature [9]. These knees in valgus HKA are influenced by complex morphological factors that extend beyond coronal plane alignment, including lateral femoral and tibial bone deficiencies, external rotation deformities of the femur and tibia, and secondary femoral metaphyseal remodeling [57]. Soft tissue alterations are also prominent, particularly contractures of the lateral soft tissues. As arthritic deformity progresses, secondary attenuation of the medial collateral ligament may occur [58]. When MA is applied to these cases, significant interventions are likely necessary, often requiring valgus resections or extensive lateral collateral ligament, iliotibial band, and posterolateral soft tissue releases. Therefore, a deeper understanding of these regional distributions is critical.

### Limitations

This review has several limitations that merit acknowledgment. Firstly, while our study expanded the dataset to include 29 articles, only a few studies in Europe and Asia had analyzed healthy populations; many countries/regions remain unrepresented in the literature regarding lower limb alignment distribution. Nevertheless, the current analysis provides valuable insights into the universality of the CPAK system and distinct regional variations in coronal plane phenotypes. Secondly, we treated population samples from the same country/region as homogeneous groups for analysis, while different regions within the same country can exhibit distinct lower limb alignment distributions. Future studies should consider regional variations within countries to enhance the accuracy of comparisons. Thirdly, this study does not explicitly address ethnic or gender-related variations. Existing evidence strongly suggests that ethnic and gender differences play a significant role in knee phenotypes [41, 53]. We anticipate that future research will increasingly report on CPAK distribution variations across diverse populations, enabling more robust comparisons stratified by ethnicity and gender.

## Conclusions

This study identified substantial differences in the distribution of CPAK types among arthritic knees across countries in North America, Europe, Australia, Asia, and Africa. For healthy knees, no substantial difference was found. These findings underscore the universality of the CPAK system and the critical importance of preoperative evaluation in patients undergoing TKA. By deepening their understanding of the phenotypic variability within their patient populations, orthopaedic surgeons can adopt a more individualized approach to TKA, potentially leading to more consistent and effective improvements in clinical outcomes.

## Data Availability

The datasets used or analyzed during the current study are available from the corresponding author on reasonable request.

## Abbreviations

CPAK: Coronal Plane Alignment of the Knee
PRISMA: Preferred Reporting Items for Systematic Reviews and Meta-Analyses
HKA: Hip-knee-ankle angle
TKA: Total knee arthroplasty
JLO: Joint line obliquity
aHKA: Arithmetic hip-knee-ankle angle
MPTA: Medial proximal tibial angle
LDFA: Lateral distal femoral angle
BMI: Body mass index
mHKA: Mechanical hip-knee-ankle angle
KA: Kinematically alignment
AA: Anatomical axis
aMA: Adjusted mechanical alignment
rKA: Restricted kinematic alignment
IKA: Inverse kinematic alignment
